# Post-Traumatic Sequelae and Their Associated Factors: A Cross-Sectional Study in the Northern Mountainous Region of Vietnam

**DOI:** 10.3390/ijerph22060905

**Published:** 2025-06-06

**Authors:** Nguyen The Diep, Tran The Hien

**Affiliations:** 1 Department of Traumatology, Thai Binh University of Medicine and Pharmacy, Thai Binh 06100, Vietnam; 2 Center for Disease Control & Prevention, Dien Bien Phu City 380100, Vietnam; hientranytb@gmail.com

**Keywords:** injury, post-traumatic sequelae, post-traumatic stress disorder

## Abstract

Background: Post-traumatic sequelae have many negative impacts on the health and quality of life of patients, especially for those groups at a high risk of exposure to injuries. Therefore, this study was conducted to identify some factors related to post-traumatic sequelae in people in a mountainous province in Northern Vietnam. Materials and Methods: A cross-sectional study was conducted with 228 residents from two communes (Pom Lot and Thanh Minh) in Dien Bien province. The participants had all experienced an injury from any cause within the year preceding the study. The post-traumatic sequelae and potential associated factors were assessed. Result: The rate of post-traumatic sequelae among the study participants was 62.3%. An older age (>40), belonging to an ethnic minority group, a short treatment duration, and a lack of family and social support were factors associated with an increased risk of post-traumatic sequelae. Conclusions: Post-traumatic stress disorder is a significant health burden for people in the mountainous regions of Northern Vietnam. This study identified vulnerable groups, particularly the elderly, ethnic minorities, and those with inadequate treatment or social support. The results highlight the urgent need for targeted interventions and focused support policies to reduce the consequences of post-traumatic stress disorder for this high-risk group.

## 1. Introduction

Post-traumatic sequelae is a medical term used to describe the long-lasting or delayed consequences of an injury. These consequences can manifest in many forms, including both physical and psychological [1]. Today, the occurrence of numerous adverse events in life has led society to pay more attention to the negative impacts of post-traumatic sequelae on health and quality of life. Especially in Western countries, the prevalence of wars, genocides, natural disasters, human-caused disasters, and terrorist attacks has further increased this concern [1].

As mentioned, post-traumatic sequelae not only relate to the physical limitations that patients experience but also affect mental health. From empirical studies, the close relationship between mental sequelae, specifically, post-traumatic stress disorder (PTSD), and physical health issues has been increasingly clarified through patient reports and objective assessments of the healthcare system. This relationship is demonstrated in various aspects, including healthcare service utilization behavior, patient quality of life, somatization syndrome (psychological problems manifesting as physical symptoms such as headaches and unexplained abdominal pain), acquired pathologies, and early mortality rates. Furthermore, this relationship is more pronounced in men than in women. It can be said that post-traumatic stress plays a significant role in the link between injury and physical health problems [2,3,4].

Factors influencing post-traumatic sequelae are diverse and depend on the type of sequelae left by the injury. For patients with PTSD, the most commonly cited related factors in studies are older age, female gender, severity of the injury, history of depression, and lack of support from family, friends, and society [5,6]. Meanwhile, for injuries that leave physical sequelae, a review study on 10 countries worldwide has shown that factors closely associated with post-traumatic sequelae include older age (>40 years), pre-injury health status, and mental health issues such as PTSD or depression [7]. While older age is recognized as a general risk factor for post-traumatic sequelae, studies specifically investigating this association among adults over 40 in the unique socio-cultural and geographical context of Vietnam’s northern mountainous regions are notably limited.

In the northern mountainous provinces of Vietnam, due to socio-economic and cultural limitations, post-traumatic sequelae from accidents and injuries have become a burden. Moreover, a shorter treatment duration (≤1 week) for injuries is associated with an increased likelihood of experiencing post-traumatic sequelae, and also, a lack of family and social support is associated with an increased likelihood of experiencing post-traumatic sequelae. Identifying factors related to post-traumatic sequelae will provide important scientific evidence, helping policymakers, authorities, and communities to develop practical and effective solutions to protect health and improve the quality of life for people in highland areas. In this context, we aimed to describe the prevalence of post-traumatic sequelae among residents who experienced injuries within the past year in two communes of Dien Bien province, a northern mountainous region of Vietnam.

Key Points

- This study provides specific data on the prevalence of post-traumatic sequelae and its associated factors among residents of a northern mountainous province in Vietnam (Dien Bien province).
- It identifies key factors significantly associated with an increased risk of post-traumatic sequelae in this specific population: an older age (>40), belonging to an ethnic minority group, a short treatment duration (≤1 week), and a lack of family and social support.
- These findings offer crucial, context-specific evidence that can inform the development of targeted public health interventions, support policies, and resource allocation aimed at mitigating post-traumatic sequelae for vulnerable groups in similar settings in Vietnam.

## 2. Materials and Methods

### 2.1. Study Design and Setting

A cross-sectional study was conducted in two communes, Pom Lot and Thanh Minh, in Dien Bien province, from June 2024 to August 2024.

### 2.2. Study Subjects

The study population consisted of residents living in two communes, Pom Lot and Thanh Minh, who had experienced injuries from any cause within one year prior to the study.

❖ Inclusion criteria: People living and working in the two study communes for at least one year. Individuals were included if they did not have a documented history of chronic, pre-existing neuropsychiatric disorders (unrelated to the injury sustained within the last year) that would impair their ability to accurately recall events or participate coherently in the interview. This criterion does not exclude neuropsychiatric sequelae developed as a consequence of the recent injury. People who agreed to participate in the study.
❖ Exclusion criteria: People who experienced injuries too long ago, making it unsuitable to assess sequelae. People with underlying medical conditions or health issues that could affect post-traumatic sequelae. People unable to comprehend or answer questions.

People who had experienced an injury from any cause within one year prior to the study. An injury was defined as meeting at least one of the following criteria: lost at least 1 day of work or school; required medical care from a medical facility or healthcare professional; were unable to participate in daily activities such as personal hygiene, bathing, laundry, sweeping, cleaning, etc., for at least 1 day.

### 2.3. Sample Size and Sampling Method

Entire sampling method: All residents living in two communes, Pom Lot and Thanh Minh, who experienced injuries from any cause within one year prior to the study, were selected. In reality, we selected 228 residents who met the inclusion and exclusion criteria to participate in the study.

### 2.4. Measurements

Injury: In this study, individuals were assessed as having an injury if they met one of the following criteria:

- Lost at least 1 day of work or school.
- Required medical care from a medical facility or healthcare professional.
- Were unable to participate in daily activities such as personal hygiene, bathing, laundry, sweeping, cleaning, etc., for at least 1 day.

Assessment of post-traumatic sequelae: The assessment of whether individuals suffered from post-traumatic sequelae was conducted by medical specialists in the field of trauma, combined with a review of patient medical records at the time of the injury.

○ Dependent Variable:

Post-traumatic sequelae: Defined as the presence of any long-lasting or delayed physical or psychological consequences of an injury that occurred within one year prior to the study. The assessment of whether individuals suffered from post-traumatic sequelae was conducted by medical specialists in the field of trauma, combined with a review of patient medical records at the time of the injury. Participants were categorized as “Yes” (experiencing sequelae) or “No” (not experiencing sequelae).

○ Independent Variables:

Socio-demographic characteristics: age groups (≤40 years and >40 years); sex (male or female); area (rural or urban, based on the participant’s residence in Pom Lot and Thanh Minh communes); ethnic groups (Kinh or ethnic minorities (specifically, Muong, Thai, H’Mong in this study population); occupation (manual labor, mental labor, or others (retired, learners, and children)).

○ Injury-related characteristics:

Injury: Assessed if the individual met one of the following criteria within one year prior to the study: lost at least 1 day of work or school, required medical care from a medical facility or healthcare professional, or were unable to participate in daily activities for at least 1 day; causal (of injury): categorized as “By yourself” or “External influences”; place of injury: categorized as “Indoors” or “Outdoors”; type of injury accident: categorized as “Traffic accident”, “Fall”, or “Others” (occupational and social accidents); health and support factors; drinking (alcohol): Categorized as “No” or “Yes” based on self-report of alcohol consumption; social support: assessed based on participant self-report regarding support received from family and society post-injury, categorized as “No” or “Yes”; first aid time (minute): time from injury to receiving first aid, categorized as ≤30 min or >30 min; time of treatment (week): duration of medical treatment for the injury, categorized as >1 week or ≤1 week; need assistance: whether the participant required assistance for daily activities post-injury, categorized as “Yes” or “No”.

### 2.5. Methods of Data Collection

Data were collected through interviews with residents using a pre-designed questionnaire consisting of two parts: general information about the residents and information about their injury status and any post-traumatic sequelae experienced in the past year.

### 2.6. Statistical Analysis

Data were cleaned by checking missing data before being entered into the database using Epidata software. The data continued to be cleaned for outliers and illogical data and were converted into an SPSS version 27.0 file for analysis. Both descriptive and inferential statistics were used. If data are normally distributed, means and standard deviations ($\bar{X}$ ± SD) were for continuous variables. Percentages were presented for nominal variables.

### 2.7. Ethical Consideration

The study was approved by the Ethics Council of Thai Binh University of Medicine and Pharmacy according to Decision No. 224/Ethics Council 10 January 2024. During the survey, participants were verbally informed about the study, that their participation was voluntary, that they had the right to withdraw at any point, and that data would be confidential. All the respondents were anonymous in the study.

## 3. Results

The data in Table 1 shows a fairly even distribution of the study sample between the under 40 and over 40 age groups. However, there is a significant gender imbalance. In terms of area, rural areas have a slight majority compared to urban areas. Notably, ethnic minorities represent an overwhelming majority, in contrast to only 24.6% who are Kinh. The predominant occupation is manual labor.

Table 2 shows a difference in the prevalence of post-traumatic sequelae across various characteristic groups. Individuals under 40 years old and females have a higher rate of sequelae. The same applies to people of Kinh ethnicity and those living in rural areas. Manual laborers and individuals with injuries caused by external influences or occurring indoors have a higher risk. Notably, non-drinkers show a higher rate of sequelae than drinkers.

Table 3 presents the relationship between sociodemographic characteristics and the prevalence of post-traumatic sequelae. The results show that an age over 40, Kinh ethnicity, and a treatment duration less than one week are factors that increase the risk of post-traumatic sequelae in the study population.

## 4. Discussion

Post-traumatic sequelae are a significant public health challenge. The consequences caused by post-traumatic sequelae are not only physical impairments but also mental disorders that occur in victims, significantly affecting survivors [8]. Our study, conducted on 228 residents in Dien Bien province—a mountainous region in Northern Vietnam—who had experienced injuries (or trauma) within one year prior to the study, showed that the majority of the injury victims (or trauma victims) in the study area were male (62.7%), belonged to ethnic minority groups (75.4%), and had primarily manual labor occupations (78.4%). These findings are consistent with those of previous studies [9,10,11]. This is understandable as males are a group that often engage in activities with high injury risks, such as heavy labor, construction, and transportation. Meanwhile, ethnic minorities may lack awareness of safety in daily life and work, leading to manual labor practices that carry many potential injury risks.

We found that individuals over 40 years old had a higher risk of post-traumatic sequelae compared to those under 40 (AOR = 2.6; 95%CI: 1.3–5.3). This is consistent with the natural aging process of the body, where the recovery capacity decreases with age. Tissues, bones, and muscles become weaker and lose elasticity, resulting in longer recovery times after injuries and a higher risk of sequelae. Age is also a related factor reported in Ihori Kobayashi’s study (2020) [12].

In addition to age, individuals from ethnic minority groups (Muong, Thai, or H’Mong) were also at a higher risk of post-traumatic sequelae compared to the Kinh majority (AOR = 2.5; 95%CI: 1.0–6.0). This is mainly due to difficult economic conditions, nutritional deficiencies, and limited access to healthcare services, which reduce the body’s recovery ability after injuries and increase the risk of sequelae [13,14]. Additionally, ethnic minority groups may have traditional living habits that increase the risk of injuries, especially severe injuries with a high rate of sequelae.

Current treatment methods for sequelae emphasize patient perseverance as a way to prevent post-traumatic stress disorder (PTSD) and limit other mental complications, requiring longer actual treatment times, even after patients have stabilized physically [15]. In our study, patients with treatment durations of one week or less had a higher risk of post-traumatic sequelae than those treated for more than one week (AOR = 5.1; 95%CI: 2.2–11.9). Short treatment durations may result in patients not receiving adequate follow-up and rehabilitation after discharge, leading to sequelae that are not detected and treated promptly. Additionally, patients with short treatment durations often return to work too early, before their bodies have fully recovered, increasing the risk of complications and sequelae.

The support factor (both from family and society), with an adjusted OR of 5.7 and a 95% confidence interval ranging from 2.8 to 11.4, shows that this factor is closely associated with post-traumatic sequelae. This result is consistent with findings from previous studies in Ethiopia [11], the United States [16], and Vietnam [17,18]. Possible explanations for this association include that family and social support can make patients feel reassured during treatment, motivated to overcome difficulties, and compliant with treatment regimens, thereby reducing the risk of post-traumatic sequelae.

### Strengths and Limitations of the Study

This study provides valuable insights into post-traumatic sequelae in a specific and often underserved population within the northern mountainous region of Vietnam. It identifies key local associated factors (older age, ethnic minority status, short treatment duration, and lack of family/social support), which can inform targeted public health interventions. The study employed clear inclusion and exclusion criteria and utilized adjusted odds ratios in the analysis to account for potential confounders.

Several limitations of this study should be mentioned. First, the cross-sectional design of the study means we can only identify associations and not establish causality. The findings are based on a sample from two communes in Dien Bien province and may not be generalizable to all mountainous regions in Vietnam or other populations. Moreover, there is a potential for recall bias, as data on injuries were collected for events occurring within the past year. While post-traumatic sequelae were assessed by medical specialists, the lack of detailed reporting on specific standardized assessment tools for all types of sequelae (beyond the general definition) could be a limitation. On the other hand, the sampling method, described as selecting all residents who met criteria, might be better characterized as a consecutive or purposive sampling of eligible individuals encountered, which could introduce selection bias.

## 5. Conclusions

In summary, this study indicated that post-traumatic sequelae are a significant disease burden for residents living in the northern mountainous provinces of Vietnam, especially among the elderly, ethnic minorities, and those with limited family and social support after injury. This highlights the need for a shift in focus within injury and trauma-related projects and support policies to specifically target these populations.

Post-traumatic sequelae are a significant disease burden in the northern mountainous provinces of Vietnam, particularly affecting the elderly, ethnic minorities, and those with limited support. Future research should consider longitudinal study designs to more definitively establish causal pathways between identified risk factors and post-traumatic sequelae. Moreover, intervention studies are warranted, focusing on developing and evaluating culturally appropriate support programs for high-risk groups, such as ethnic minorities and the elderly, in these mountainous regions. This could include enhancing community-based support systems and improving access to rehabilitation services.

## Figures and Tables

**Table 1:** Socio-demographic characteristics of study participants in the northern mountainous region of Vietnam, 2024 (*n* = 228).

Characteristics		Frequency	Percentage
Age groups	≤40	108	47.4
	>40	120	52.6
Sex	Male	143	62.7
	Female	85	37.3
Area	Rural	121	53.1
	Urban	107	46.9
Ethnic groups	Kinh	56	24.6
	Minorities *	172	75.4
Occupation	Manual labor	181	78.4
	Mental labor	18	7.9
	Others **	29	12.7

Minorities *: Muong, Thai, or H’Mong. Others **: retired, student, or child.

**Table 2:** Prevalence of post-traumatic sequelae of study participants in the northern mountainous region of Vietnam, 2024 (*n* = 228).

Characteristics		Post-Traumatic Sequelae	
		No *n *(%)	Yes *n* (%)
Age groups	≤40	74 (68.5)	34 (31.5)
	>40	68 (56.7)	52 (43.3)
Sex	Male	84 (58.7)	59 (41.3)
	Female	58 (68.2)	27 (31.8)
Area	Rural	79 (65.3)	42 (34.7)
	Urban	63 (58.9)	44 (41.1)
Ethnic groups	Kinh	42 (75.0)	14 (25.0)
	Minorities *	100 (58.1)	72 (41.9)
Occupation	Manual labor	113 (62.4)	68 (37.6)
	Mental labor	10 (55.6)	8 (44.4)
	Others **	19 (65.5)	10 (34.5)
Causal	By yourself	97 (59.1)	67 (40.9)
	External influences	45 (70.3)	19 (29.7)
Place of injury	Indoors	56 (69.1)	25 (30.9)
	Outdoors	86 (58.5)	61 (41.5)
Drinking	No	119 (66.1)	61 (33.9)
	Yes	23 (47.9)	25 (52.1)

Minorities *: Muong, Thai, or H’Mong. Others **: retired, student, or child.

**Table 3:** Factors associated with post-traumatic sequelae of study participants in the northern mountainous region of Vietnam, 2024 (*n* = 228).

Variables		Post-Traumatic Sequelae			
		No *n* (%)	Yes *n* (%)	COR (95% CI)	AOR (95% CI)
Age groups	≤40	74 (68.5)	34 (31.5)	1	1
	>40	68 (56.7)	52 (43.3)	1.7 (1.0–2.9) *	2.6 (1.3–5.3) *
Sex	Male	84 (58.7)	59 (41.3)	1	1
	Female	58 (68.2)	27 (31.8)	0.7 (0.4–1.2)	0.6 (0.3–1.5)
Area	Rural	79 (65.3)	42 (34.7)	1	1
	Urban	63 (58.9)	44 (41.1)	1.3 (0.8–2.2)	0.6 (0.3–1.5)
Ethnic groups	Kinh	42 (75.0)	14 (25.0)	1	1
	Minorities *	100 (58.1)	72 (41.9)	2.2 (1.1–4.2) *	2.5 (1.0–6.0) *
Occupation	Manual labor	113 (62.4)	68 (37.6)	1	1
	Mental labor	10 (55.6)	8 (44.4)	1.3 (0.5–3.5)	2.4 (0.6–9.1)
	Others **	19 (65.5)	10 (34.5)	0.9 (0.4–2.0)	0.9 (0.3–2.7)
Causal	By yourself	97 (59.1)	67 (40.9)	1	1
	External influences	45 (70.3)	19 (29.7)	0.6 (0.3–1.1)	0.5 (0.2–1.1)
Place of injury	Indoors	56 (69.1)	25 (30.9)	1	1
	Outdoors	86 (58.5)	61 (41.5)	1.6 (0.9–2.8)	1.7 (0.7–3.8)
Drinking	No	119 (66.1)	61 (33.9)	1	1
	Yes	23 (47.9)	25 (52.1)	2.1 (1.1–4.0) *	1.1 (0.4–2.8)
Type of injury accident	Traffic accident	34 (54.8)	28 (45.2)	1	1
	Fall	39 (60.9)	25 (39.1)	0.8 (0.4–1.6)	2.1 (0.8–6.1)
	Others ***	69 (67.6)	33 (32.4)	0.6 (0.3–1.1)	1.1 (0.5–2.9)
Social support	No	75 (67.0)	37 (33.0)	1	1
	Yes	67 (57.8)	49 (42.2)	1.5 (0.9–2.5)	1.2 (0.6–2.5)
First aid timent (minute)	≤30	116 (64.1)	65 (35.9)	1	1
	>30	26 (55.3)	21 (44.7)	1.4 (0.8–2.8)	1.3 (0.6–3.1)
Time of treatment (week)	>1	60 (47.6)	66 (52.4)	1	1
	≤1	82 (80.4)	20 (19.6)	4.5 (2.5–8.2) ***	5.1 (2.2–11.9) ***
Need assistance	Yes	33 (36.7)	57 (63.3)	1	1
	No	109 (79.0)	29 (21.0)	6.5 (3.6–11.7) ***	5.7 (2.8–11.4) ***

Minorities *: Muong, Thai, or H’Mong. Others **: retired, student, or child. Others ***: occupational and social accidents. COR: crude odds ratio; AOR: adjusted odds ratio; * *p* < 0.05, ** *p* < 0.01, *** *p* < 0.001.

## Data Availability

The datasets underlying the results of this study are available from the corresponding author upon reasonable request. Requests to access these datasets should be directed to diepnguyentheytb@gmail.com.

## Abbreviations

The following abbreviations are used in this manuscript:

AOR	Adjusted odds ratio
COR	Crude odds ratio
PTSD	Post-traumatic stress disorder

## References

[1] Kapfhammer H.P. (2018). Acute and long-term mental and physical sequelae in the aftermath of traumatic exposure—Some remarks on “the body keeps the score”. Psychiatr. Danub..

[2] Pacella M.L., Hruska B., Delahanty D.L. (2013). Delahanty The physical health consequences of PTSD and PTSD symptoms: A meta-analytic review. J. Anxiety Disord..

[3] Wooldridge J.S., Herbert M.S., Dochat C., Afari N. (2021). Understanding relationships between posttraumatic stress disorder symptoms, binge-eating symptoms, and obesity-related quality of life: The role of experiential avoidance. Eat Disord..

[4] Sommer J.L., Reynolds K., El-Gabalawy R., Pietrzak R.H., Mackenzie C.S., Ceccarelli L., Mota N., Sareen J. (2021). Associations between physical health conditions and posttraumatic stress disorder according to age. Aging Ment. Health.

[5] Bonsaksen T., Heir T., Schou-Bredal I., Ekeberg Ø., Skogstad L., Grimholt T.K. (2020). Post-Traumatic Stress Disorder and Associated Factors during the Early Stage of the COVID-19 Pandemic in Norway. Int. J. Environ. Res. Public Health.

[6] Armenta R.F., Rush T., LeardMann C.A., Millegan J., Cooper A., Hoge for the Millennium Cohort Study Team (2018). Factors associated with persistent posttraumatic stress disorder among U.S. military service members and veterans. BMC Psychiatry.

[7] Alharbi R., Mosley I., Miller C., Hillel S., Lewis V. (2019). Factors associated with physical, psychological and functional outcomes in adult trauma patients following Road Traffic Crash: A scoping literature review. Transp. Res. Interdiscip. Perspect..

[8] Maas A.I.R., Menon D.K., Manley G.T., Abrams M., Åkerlund C., Andelic N., Aries M., Bashford T., Bell M.J., Bodien Y.G. (2022). Traumatic brain injury: Progress and challenges in prevention, clinical care, and research. Lancet Neurol..

[9] Liu H., Yi T. (2024). Risk factors for psychiatric disorders following traumatic brain injury: A multivariate logistic regression analysis. Front. Psychiatry.

[10] Kassaye A., Demilew D., Fanta B., Mulat H., Ali D., Seid J., Mulugeta A., Dereje J. (2023). Post-traumatic stress disorder and its associated factors among war-affected residents in Woldia town, North East Ethiopia, 2022; community based cross-sectional study. PLoS ONE.

[11] Melkam M., Tinsae T., Andualem F., Nakie G. (2023). Post-traumatic stress disorder and associated factors among adults exposed to stress in Ethiopia: A meta-analysis and systematic review. SAGE Open Med..

[12] Kobayashi I., Hatcher M., Wilson C., Boadi L., Poindexter M., Allard J.S., Polston E.K. (2020). Impacts of sex and the estrous cycle on associations between post-fear conditioning sleep and fear memory recall. Behav. Brain Res..

[13] Nguyen K.T., Khuat O.T.H., Ma S., Pham D.C., Khuat G.T.H. (2012). Barriers to health care access among ethnic minorities in remote areas in Vietnam: A qualitative study. Glob. Health Action.

[14] Nguyen P.H., Nguyen H.V., Gonzalez-Casanova I., Le B.M., Pham H.T., Truong T.V., Ramakrishnan U. (2016). Multisectoral nutrition-sensitive interventions reduced child stunting and maternal underweight and improved food security and diet diversity: A cluster-randomised controlled trial in Vietnam. J. Nutr..

[15] Schrader, Ross A. (2021). A Review of PTSD and Current Treatment Strategies. Mo. Med..

[16] Tsai, Shen J. (2017). Exploring the Link Between Posttraumatic Stress Disorder and inflammation-Related Medical Conditions: An Epidemiological Examination. Psychiatr. Q..

[17] Hoi D.D., Son H.V., My N.T.D., Vu G.T. (2019). Factors affected the psychological trauma of children living in incomplete families—The concern in Vietnamese school counseling. Eur. J. Educ. Res..

[18] Thach C.T., Nguyen T.H.M., Vo T.H.N., Vo T.C.N., Nguyen D.N.Q., Nguyen H.T., Tang T.N., Nguyen T.H., Do V.T., Truong Q.B. (2022). Post-traumatic stress disorder, anxiety, depression and related factors among COVID-19 patients during the fourth wave of the pandemic in Vietnam. Int. Health.

